# Towards appropriate polypharmacy in older cardiovascular patients: How many medications do I have to take?

**DOI:** 10.1002/clc.23304

**Published:** 2019-12-11

**Authors:** Magali P. A. Disdier Moulder, Abby K. Hendricks, Narith N. Ou

**Affiliations:** ^1^ Department of Pharmacy Mayo Clinic Rochester Minnesota

**Keywords:** deprescribing, geriatrics, pharmacology

## Abstract

**Background:**

Polypharmacy in older adults leads to increased risks of side effects and drug‐drug interactions, affecting their health outcomes and quality of life. Deprescribing, the act of simplifying medication regimens, is challenging due to the lack of consensus guidelines.

**Hypothesis:**

To offer some guidance on managing medication regimens for older cardiovascular patients.

**Methods:**

We reviewed the most recent pertinent guidelines and literature.

**Results:**

This review provides practical considerations for appropriate prescribing in the older population with cardiovascular disease in order to strike a balance between unnecessary or harmful medications and therapies with proven long‐term benefits.

**Conclusion:**

On‐going dialogue between healthcare providers and patients allows close monitoring of medication effectiveness and prevention of side effects. Medication regimens require individualization, as patients' goals of care change with advancing age.

**ABBREVIATIONS**
ACC: American College of Cardiology
ACE‐i: Angiotensin Converting Enzyme inhibitor
ACS: Acute Coronary Syndrome
AF: Atrial Fibrillation
AHA: American Heart Association
AKI: Acute Kidney Injury
ARB: Angiotensin II Receptor Blocker
ARNI: Angiotensin Receptor‐Neprilysin Inhibitor
ASCVD: AtheroSclerotic CardioVascular Disease
AV block: AtrioVentricular node block
CAD: Coronary Artery Disease
CCB: Calcium Channel Blocker
CDC: Center for Disease Control (and Prevention)
CKD: Chronic Kidney Disease
DAPT: Dual Anti Platelet Therapy
DDI: Drug‐Drug Interaction
DM: Diabetes Mellitus (2; type 2)
DOAC: Direct Oral AntiCoagulant
DPP‐4 i: (Dipeptidyl Peptidase‐4) inhibitor
DVT: Deep Vein Thrombosis
eGFR: estimated Glomerular Filtration Rate
HFpEF: Heart Failure with preserved Ejection Fraction (left ventricular ejection fraction above 50%)
HFrEF: Heart Failure with reduced Ejection Fraction (left ventricular ejection fraction less than 40%)
HTN: HyperTension
LVEF: Left Ventricular Ejection Fraction
NSAID: Non‐Steroidal Anti‐Inflammatory Drug
NSTEMI: Non ST Elevation Myocardial Infarction
PE: Pulmonary Embolism
PPI: Proton pump inhibitor
RAAS‐i: Renin‐Angiotensin‐Aldosterone System inhibitor
SAMS: Statin‐Associated Muscle Symptom
SBP: Systolic Blood Pressure
SGLT‐2: (Sodium Glucose CoTransporter‐2) inhibitor
STEMI: ST Elevation Myocardial Infarction

## 1 INTRODUCTION

Polypharmacy, defined as concomitant use of five or more daily medications, becomes increasingly common as patients advance in age.^1^ The definition of older adult varies in the literature, ranging from any patient over 65 years of age, per Center for Disease Control (and Prevention) (CDC) recommendations, up to patients greater than 70 or 75 years based on American College of Cardiology/American Heart Association (ACC/AHA) guidelines for primary prevention and Non ST Elevation Myocardial Infarction (NSTEMI) management, respectively.^2^, ^3^, ^4^ Another consideration when managing pharmacotherapy for older adults is the potential discrepancy between chronologic and physiologic age, with the latter taking into account comorbidity burden. Management of multiple chronic conditions, each driven by disease specific treatment guidelines, is a major contributor to polypharmacy.^1^, ^5^

When assessing the appropriateness of pharmacotherapy in the geriatric population several important issues need consideration. First, treatment guidelines were established in younger populations, and thus data showing benefits in older adults may remain sparse.^6^ Second, aging results in physiologic changes that have the potential to impact drug clearance and third cognitive decline may limit a patient's ability to manage complex medication regimens.^3^, ^7^, ^8^ Finally, polypharmacy increases the risk of drug‐drug Interactions (DDI). All of these factors can predispose older adults to drug accumulation and severe adverse effects. On the other hand, under‐treatment, when therapy is withheld despite data clearly showing long term benefit, is also common in the older population.^3^, ^9^

Thus it is necessary to maintain a delicate balance between over‐treatment leading to problematic polypharmacy secondary to medication use in the absence of strong indications,^5^ and under‐treatment when therapy is not prescribed despite a clear benefit. The goal is “appropriate polypharmacy,” utilizing optimized medication regimens, according to best clinical practices shown to improve patient outcomes. It is imperative that clinicians regularly review the appropriateness of medications in both the clinic and acute care settings. Furthermore, involvement of patients and families in the shared‐decision making process will help to ensure their understanding of changes in medical management and improve patients' quality of life.

The American Geriatric Society (AGS) and the National Institute on Aging (NIA) developed tools to identify medications that are potentially inappropriate for older adults, delineate priorities and guiding principles for safe medication use, while promoting research on deprescribing practices in older populations.^10^, ^11^, ^12^ The recently updated AGS Beers Criteria compiles a comprehensive list of medications potentially inappropriate in older patients.^10^ Other tools are found in the literature: The STOPFrail criteria applies more specifically to the most vulnerable and frail patients; the STOPP/START criteria describes the use of software to aid in detecting inappropriate medications.^13^, ^14^ The Pharmacist's Letter also provides a number of tool kits and guides to deprescribing.^15^ In this article we aim to provide some practical considerations on deprescribing, dose reduction, and drug selection in the older adult population.

### 1.1 Primary prevention

Primary prevention for cardiovascular disease in older adults focuses on controlling major risk factors including hypertension, hyperlipidemia, and diabetes mellitus.^2^ Chronic kidney disease becomes prevalent with advancing age, and often guides selection and dosing of pharmacotherapy.

#### 1.1.1 Blood pressure control

Current hypertension guidelines recommend mean blood pressure of less than 130/80 mmHg in patients over 79 years old.^16^ However, resistant hypertension is common in the older population, in which 79% of men and 85% of women over 75 do not reach their blood pressure goals.^17^ This commonly results in multiple agents being prescribed or titrated rapidly in an attempt to control hypertension, increasing the risks of drug interactions, orthostatic hypotension and falls when the medications achieve steady state. Generally, medication review or dose adjustment should be considered whenever patients experience dizziness, low systolic blood pressure (SBP < 85 mmHg) or bradycardia (Heart Rate <55 bpm). In the absence of compelling indications, hypertension agents should be started one at a time, at low doses and up‐titrated over several weeks to the maximum tolerated dose before adding a different agent. This approach also has the advantage of decreasing pill burden. Monitoring parameters include vital signs, renal function and electrolytes as appropriate (see Table 1), noting that some medications (eg, amlodipine, lisinopril) reach their full effect after several weeks. It is best to avoid atenolol as it is a relatively less effective antihypertensive agent and may accumulate in older adults with renal dysfunction.

**Table 1:** Common cardiovascular medications and how to de‐prescribe: DDIs, Drug‐Drug Interactions; eGFR, estimated Glomerular Filtration Rate; ER, Extended Release; Hx, History; inh., inhibitor; INR, International Normalized Ratio; NTE, Not To Exceed; renal Cl, renal clearance; OAC, Oral Anti‐Coagulant; PRN, as needed; SL NTG, Sub Lingual Nitroglycerin

Medication Class/Name	Disease state	Deprescribe or hold^a^ Yes/No	Why	When	Comments
Aspirin	Primary prevention	Yes	Bleeding risk	>75 years old and NO ASCVD	Questionable benefit
	Post ACS	Yes	Bleeding risk if DAPT+ OAC	Hold aspirin while on P2Y12 antagonist and OAC	Reassess once P2Y12 antagonist completed
	Stable CAD	Yes	Bleeding risk	While on DOAC	Rivaroxaban enough for CAD and AF^31^
P2Y12 antagonist (Clopidogrel/ Ticagrelor)	Post ACS	No (See comments)	Thrombosis risk	Increased thrombosis risk after ACS	Reassess 1 year after ACS or defined duration Prasugrel not recommended in patients 75 or older
Statins	Primary prevention	Yes	SAMS risk, pill burden	Limited life expectancy	3 to 5 years therapy to show benefits
	Post ACS Secondary prevention	No (see comments)	Pleiotropic benefit	Important right after ACS for pleiotropic properties	Reassess if stable CAD Use moderate intensity
Ezetimibe	CAD secondary prevention	Yes	Pill burden	Useful only for patients at high ASCVD risk or intolerant of statin	Very well tolerated Increases pill burden
PCSK9‐i	CAD secondary prevention	Yes	Parenteral Costly		Limited clinical experience
Fibrates Fish oil Niacin	CAD secondary prevention	Yes	Limited benefits Myopathies	Assess for deprescribing anytime	Prescription strength omega‐3 approved for hyper‐triglyceridemia
Thiazides diuretics	Hypertension	Yes	Limited effectiveness in CKD Increase risk of diabetes	Limited effectiveness if Renal clearance<40 mL/min	Consider alternative agents
ACE‐i, ARB	Primary prevention	Yes (see comments)	AKI, hyperkalemia	When Serum creatinine increases by 0.5 mg/dL in 24 hours or > 2.5 mg/dL in women or > 3 mg/dL in men K+ > 5 mEq/L	Reasonable use for diabetes and stable CKD.
RAAS (ACE‐i, ARB, Aldosterone antagonist)	Post ACS	Yes (see comments)	AKI, hyperkalemia		Cornerstone of therapy if no contraindication
RAAS (ACE‐i, ARB, Aldosterone antagonist, ARNI)	HFrEF LVEF<40%	Yes (see comments)	AKI hyperkalemia		Cornerstone of therapy if no contraindication Wash out 36 hours between ACE‐I and ARNI
CCB non‐dihydropyridines (diltiazem/ verapamil)	Hypertension	Yes	May worsen HFrEF symptoms (negative inotropic)	Acute HFrEF	Consider amlodipine if for hypertension Beta‐blocker preferred in ACS
	Atrial fibrillation rate control				
Beta‐Blockers (Metoprolol ER, Carvedilol, Bisoprolol)	Hypertension	Yes	Fatigue, Inferior efficacy	Fatigue, high grade AV block without pacemaker	Not recommended as first line Avoid atenolol (renal elimination)
	Post ACS	Yes (See comments)	Fatigue, bradycardia without pacemaker	3 years post ACS if LVEF>40%^30^	Cornerstone of therapy right after ACS if no contraindication
	HFrEF			Discuss goals of care with patient	HFpEF: only to control heart rate or SBP
Nitrates	CAD, angina	Yes	No mortality benefits; Symptom relief	Free of angina	Require daily nitrate‐free interval. Keep SL NTG PRN for angina
Digoxin	Atrial fibrillation	Yes	Not first line for chronic use, narrow therapeutic window	Toxicity common with chronic use; AKI; significant DDIs	Use if BB or CCBs not tolerated Short term use
	HFrEF				Narrow therapeutic window 0.5‐0.9 ng/mL Stopping therapy may worsen heart failure symptoms
Loop diuretics (Furosemide, Torsemide, Bumetanide)	HFrEF, HFpEF	No (see comments)	Cornerstone of fluid management		Reduce dose when resolution of edema. If diuretic resistance switch to more potent agent .Furosemide‐NTE 600 mg/day^30^ . Furosemide 40 mg equiv. 20 mg torsemide equiv. 1 mg bumetanide^39^
Metolazone	HFrEF, HFpEF and diuretic resistance	Yes	AKI and hypokalemia	Long acting, usually given 2 to 3 doses per week	Give 30 minutes before loop diuretic
DOACS (Apixaban, Rivoroxaban, Dabigatran)	Non‐valvular Atrial Fibrillation	Yes	Bleeding risk	AKI, advanced CKD;. Significant DDIs may require reduced dosing or stopping	May be preferred over warfarin Apixaban preferred agent in older adult
VKA Warfarin	Atrial Fibrillation	Yes	Bleeding risk	Significant DDIs Right heart failure decreases clearance	Close INR monitoring in setting of DDI, poor oral intake, hepatic congestion.
Metformin	Diabetes	Yes	Lactic acidosis risk	Caution if renal Cl < 45 mL/min STOP if renal Cl < 30 mL/min	1st line Low risk of hypoglycemia
Sulfonylureas	Diabetes	Yes	Hypoglycemia risk		Consider newer agent
SGLT‐2 inh	Diabetes	Yes	Genito‐urinary infection and possible diabetic ketoacidosis	AKI Decrease or stop in advanced CKD	Limited clinical experience in older patient
DPP‐4 inh	Diabetes	Yes	Hypoglycemia In advanced CKD	Decrease or stop in advanced CKD	Limited experience
GLP‐1 receptor agonists	Diabetes	Yes	Hypoglycemia Caution if use with concomitant insulin	Do not use if renal Cl < 30 mL/min	Parenteral agents Limited experience
Insulin	Diabetes	Yes	Complicated regimen requires manual dexterity	Simplify regimen with once daily long‐acting agent	Parenteral Complicated regimen

Abbreviations: ACE‐i, Angiotensin Converting Enzyme inhibitor; ACS, Acute Coronary Syndrome; AF, Atrial Fibrillation; AKI, Acute Kidney Injury; ARB, Angiotensin II Receptor Blocker; ARNI, Angiotensin Receptor‐Neprilysin Inhibitor; ASCVD, AtheroSclerotic CardioVascular Disease; AV, AtrioVentricular; BB: Beta‐Blocker; CAD, Coronary Artery Disease; CCB, Calcium Channel Blocker; CKD, Chronic Kidney Disease; DAPT, Dual Anti Platelet Therapy; DOAC, Direct Oral AntiCoagulant; DPP‐4, Dipeptidyl Peptidase‐4; GLP‐1, Glucagon‐Like Peptide‐1; LVEF, Left Ventricular Ejection Fraction; RAAS, Renin‐Angiotensin‐Aldosterone System; SAMS, Statin‐Associated Muscle Symptom; SBP, Systolic Blood Pressure; SGLT‐2, Sodium Glucose CoTransporter‐2 inhibitor; VKA, Vitamin K Antagonist.

a Intended as general recommendations. Assess benefits and risks for individual patient and situation.

#### 1.1.2 Hyperlipidemia

De novo hyperlipidemia management for primary prevention in older adults focuses on lifestyle modifications and thorough assessment of risk factors for AtheroSclerotic CardioVascular Disease (ASCVD) (diabetes, smoking history).^18^, ^19^ The ASCVD risk score is valid only until 79 years of age and data remain sparse for older patients. In‐depth dialogue with patients and their family members regarding the risks and benefits of therapy as well as consideration of patient preference is necessary before initiating a statin. For patients already on a statin at the age of 75, the decision of continuing or stopping statin therapy depends on tolerability, risk for a cardiovascular event, and patient preference. For a more detailed discussion, refer to the chapter on Coronary Artery Disease (CAD) and secondary prevention.

#### 1.1.3 Diabetes mellitus

According to Center for Disease Control and Prevention data, 25% of older adults have a diagnosis of diabetes mellitus.^20^ Current diabetes guidelines allow for less aggressive blood sugar control in older adults depending on co‐morbidities.^21^, ^22^, ^23^ For instance while healthy older adults may still aim for HgbA1c less than 7.5%, guidelines permit HgbA1c goals of 8% to 8.5% for patients with functional and cognitive decline. Patients' life expectancy, co‐morbidities, health literacy and ability to manage complex medication regimens should be assessed and therapy should be simplified accordingly. Metformin is an appropriate first line agent; however, alternative therapies should be considered in the case of renal disease (estimated Glomerular Filtration Rate [eGFR] <30 mL/min/1.73m^2^), liver dysfunction or heart failure due to the risk of lactic acidosis. Newer oral agents like Dipeptidyl Peptidase‐4 (DPP‐4) inhibitors and Sodium Glucose CoTransporter‐2 (SGLT‐2) inhibitors are attractive alternatives. SGLT‐2 inhibitors may be especially beneficial considering their diuretic properties and usefulness in heart failure patients, but clinical experience is still limited. Caution is warranted due to risks of hypoglycemia (DPP‐4 inhibitors), urinary and genital infections, and diabetic ketoacidosis (SGLT‐2 inhibitors). Glucagon‐Like Peptide‐1 receptor agonists are parenteral, and like insulin, require manual dexterity. If insulin is chosen, guidelines suggest a simple regimen of once‐daily fixed‐dose long‐acting insulin administered via an insulin pen. Older diabetes medications (ie, sulfonylureas, thiazolidinedione, glinides) should be avoided due to the risk of hypoglycemia or worsening of cardiovascular disease.^21^

#### 1.1.4 Miscellaneous common medications

*Aspirin*: Based on recent trials showing lack of cardiovascular risk reduction benefit along with an increased propensity for bleeding events, aspirin therapy for primary prevention of ASCVD in patients over 75 is no longer recommended by consensus guidelines.^22^, ^24^ Clinicians should regularly assess for unnecessary aspirin use and discontinue therapy when able.

*Proton pump inhibitors (PPI)*: Guidelines support the use of PPIs in patients at an increased risk for gastrointestinal bleed and prophylactic PPIs are prescribed for older adults on systemic anticoagulation, antiplatelet therapy or after a gastrointestinal bleed episode. However, recent clinical trials have proven the safety of omitting aspirin when a P2Y12 inhibitor is used in conjunction with systemic anticoagulation.,^25^, ^26^ lessening the need for a proton pump inhibitor. Moreover, the latest guidelines recommend stopping the proton pump inhibitor 2 years after a bleeding episode if no recurrence as long term use of PPIs has been associated with numerous complications including *Clostridium difficile* infection, hypomagnesemia, and aspiration pneumonia.^27^, ^28^

#### 1.1.5 Vitamins and nutritional supplements

Vitamins and supplements are commonly believed to be safe with few side effects, leading to a false sense of security with their use; however, use of these agents in conjunction with prescription medications can lead to significant drug interactions and adverse effects.^29^ Heart failure guidelines in particular, discourage the use of supplements in addition to guideline‐directed medical therapy.^30^ Despite questionable benefit, even possible harm, routine use of vitamins and supplements to prevent cardiovascular diseases remains a common occurrence. Clinicians should address the risks and benefits and recommend discontinuation of supplements without clear benefits.

### 1.2 Secondary prevention and established CAD

The treatment of acute coronary syndrome (ACS) is well established and well defined by consensus guidelines.^3^, ^9^ Despite limited enrollment in clinical trials, older adults derive mortality benefits from guideline‐recommended medications for secondary prevention after ACS; however, the benefits must be balanced with an increased risk of adverse side effects and DDIs.

*Antiplatelet Therapy: Aspirin (ASA) and P2Y12 Antagonist (eg, clopidogrel, ticagrelor)*: Aspirin therapy has a class I recommendation as a life‐long therapy after ACS as it decreases mortality and recurrence of cardiovascular events.^3^, ^9^ In the context of ACS, guidelines also recommend 12 months of dual anti platelet therapy (DAPT) using aspirin in combination with a P2Y12 inhibitor, regardless of treatment modality (medical management, percutaneous coronary intervention [PCI] or coronary artery bypass graft). However, DAPT is associated with increased bleeding risk, especially in older adults. To mitigate this risk aspirin doses should not exceed 100 mg per day. Furthermore, consider stopping the P2Y12 antagonist earlier in patients with an elevated bleeding risk. Of note, prasugrel is not recommended in patients over 75.

Triple therapy in patients requiring long‐term oral anticoagulation should be limited to the shortest possible duration. Recent trials have shown that aspirin may be safely omitted while continuing dual therapy with an anticoagulant and P2Y12 receptor antagonist.^25^, ^26^ Aspirin may be resumed once the P2Y12 inhibitor is stopped, although recent data show that rivaroxaban monotherapy is safe and adequate in patients with stable CAD and atrial fibrillation.^31^ Monitor patients closely in the first 90 days after P2Y12 discontinuation for recurrent ischemic events.^32^

*Statins*: High‐intensity statin therapy (goal LDL‐C < 70 mg/dL) is recommended after ACS for pleiotropic effects,^3^, ^9^, ^18^, ^19^ but older adults have a greater risk of statin‐associated muscle symptoms and moderate‐intensity statin therapy may be preferred.^18^, ^19^ In order to reduce the incidence of adverse effects, the dose of simvastatin in particular should not exceed 40 mg daily.^33^ In the very old (> 90 years old) with no recent ACS, statin therapy may be discontinued following a risk‐benefit discussion with the patient, noting that statin‐derived benefits are seen after 4 to 5 years of therapy.^18^, ^19^

Guidelines make provision for *ezetimibe* and *PCSK9‐inhibitors* for patients unresponsive or intolerant to statin therapy.^3^, ^9^, ^18^ Ezetimibe is very well tolerated, but discussion regarding expected benefits vs additional polypharmacy should be had prior to initiation. PCSK9‐inhibitors are powerful, parenteral, and costly anti‐lipid agents and have a limited use in older patients.^18^

*Fibrates* should usually be avoided due to limited LDL Cholesterol (LDL‐C) lowering benefits and notable adverse effects (eg, myopathies), unless indicated for triglyceride lowering. *Niacin* lacks clinical benefit and is no longer recommended.^18^ *Fish oil* supplementation has been extensively studied. While the Federal Drug Administration (FDA) allows a claim that fish oil may reduce the risk of coronary disease the agency points out the evidence is inconclusive and inconsistent.^34^ In the United States, two prescription strength formulations (ie, Lovaza* and Vascepa*) have secured indications for severe hypertriglyceridemia (≥500 mg/dL).^35^, ^36^ Fish oil supplements should be targeted for deprescribing especially if being used for primary prevention.

β*‐blockers*: Beta‐blockers such as metoprolol and carvedilol carry a class I recommendation post‐ACS per consensus guidelines and are usually started as early as 24 hours after ACS.^3^, ^9^ In the older adult, beta‐blocker therapy may contribute to cognitive impairment and fatigue, especially with highly lipophilic agents such as metoprolol, while carvedilol can lead to pronounced hypotension. In the era of postrevascularization, the long‐term benefits of beta‐blockers have been called into question; guidelines even suggest to reassess their utility at 3 years post‐ACS in patients with Left Ventricular Ejection Fraction (LVEF) >40%.^37^

*Renin‐angiotensin‐aldosterone system inhibitors (ie, Angiotensin Converting Enzyme [ACE] inhibitors, Angiotensin II Receptor Blocker [ARB], aldosterone inhibitors)*: Renin‐angiotensin‐aldosterone system (RAAS) inhibitors are a cornerstone of guideline‐directed medical therapy post‐ACS, especially if left ventricular dysfunction is present.^37^ Older adults are at higher risk of acute kidney injury, and should be closely monitored for worsening renal dysfunction and hyperkalemia. Addition of an aldosterone antagonist to either an ACE‐inhibitor or ARB should be done cautiously, while the combination of an ACE‐inhibitor and an ARB should be avoided altogether. Consider reducing the dose or a temporary hold vs stopping therapy for worsening renal dysfunction or hyperkalemia (ie, serum creatinine>2.5 mg/dL in women, 3 mg/dL in men, K+ >5 mEq/L).^30^ Avoid nephrotoxic medications like over‐the‐counter Non‐Steroidal Anti‐Inflammatory Drug (NSAIDs) or medications that can induce hyperkalemia, such as potassium‐sparing agents, trimethoprim, or potassium‐based salt substitutes.^3^, ^9^

*Nitrates*: Nitrates can relieve symptoms associated with cardiac ischemia but do not reduce mortality, in which case chronic use should be reserved for coronary vasospasm or incomplete revascularization.^3^ Long‐acting, once‐a‐day formulations cause less hypotension and are preferred. Sublingual nitroglycerin remains an important medication to have on hand for a relief of an ischemic attack.

### 1.3 Heart failure

Heart failure prevalence is as high as 13% in patients over the age of 80.^30^ Treatment guidelines for heart failure with reduced ejection fraction (HFrEF, LVEF <40%) recommend chronic use of RAAS inhibitors (ie, ACE‐i, ARB, Angiotensin Receptor‐Neprilysin Inhibitor [ARNI], aldosterone antagonists) and beta‐blockers to reduce morbidity and mortality.^30^, ^38^ However, treatments for heart failure with preserved ejection fraction (HFpEF, LVEF >50%) rely largely on fluid management with diuretics and optimization of associated comorbidities. Standard therapy for HFrEF may not provide any benefit for HFpEF but seems adequate for patients with intermediate LVEF (40%‐50%).^30^ On‐going assessment of treatment benefits and tolerability is necessary, along with revisiting goals of care.

*Diuretics*: Although loop diuretics (eg, furosemide, torsemide, bumetanide) have not demonstrated survival benefit, they remain a cornerstone of fluid management, regardless of left ventricular ejection fraction.^30^ Furosemide has highly variable oral bioavailability, and switching to a more potent agent, for example, torsemide or bumetanide, may overcome diuretic resistance and reduces pill burden.^39^ Maintenance diuretic therapy should be adjusted to maintain euvolemia with the use of intermittent metolazone as indicated (see Table 1). Thiazide agents may not be effective in the setting of low eGFR (less than 40 mL/min), and should be assessed for discontinuation.

*RAAS inhibitors*: ACE‐i/ ARBs/ARNI/aldosterone antagonists: This class of agents provides significant benefits in HFrEF; however, their role in HFpEF is limited to the management of underlying co‐morbidities (eg, hypertension, diabetes mellitus, renal dysfunction).^30^ Therapy discontinuation or pause and re‐assessment may be necessary in acute renal dysfunction, hyperkalemia or hypotension in advanced heart failure. Patients with HFrEF who tolerate an ACE‐i or ARB are highly encouraged to switch to the combination of ARB/ARNI (valsartan/sacubitril) for an additional 20% reduction in cardiovascular mortality and heart failure hospitalizations.^40^

*Beta‐blockers*: Metoprolol Extended Release, carvedilol and bisoprolol have shown benefits in HFrEF. Similar to above, their role in HFpEF is limited to treating underlying conditions (eg, atrial arrhythmias).^30^

*Digoxin*: The use of digoxin for chronic heart failure has declined as benefits appear to be limited to symptomatic relief without mortality benefit. Furthermore, appropriate management is difficult due to its narrow therapeutic window (0.5 to 0.9 ng/mL) and older adults in particular are at risk for digoxin toxicity secondary to declining renal drug clearance. In this case it is imperative that renal function be monitored frequently to minimize drug accumulation. Prior to initiating digoxin in older adults, optimization of first‐line guideline‐directed medical therapy should be performed. Discontinuation of digoxin should be considered but requires monitoring for signs of worsening symptoms as stopping therapy may be associated with poorer outcomes among patients with heart failure.^41^

On‐going goals of care discussions are paramount as patients' heart failure progresses towards advanced stages. Quality of life can be greatly improved by simplification of the medical regimen. Review and assess the tolerability of recommended therapies and eliminate adjuvant agents that can exacerbate heart failure symptoms (sotalol, dronedarone, propafenone, verapamil, diltiazem, cilostazol, metformin, thiazolidinediones, NSAIDs, etc).^42^

### 1.4 Arrhythmias, atrial fibrillation

Atrial fibrillation (AF) is a common cardiac arrhythmia seen in older adults with prevalence increasing with age.^43^ The primary goals of AF management include prevention of thromboembolic events, primarily ischemic stroke, and reduction of symptoms and hospitalizations.^43^

*Anticoagulation*: Systemic anticoagulation should be considered in all patients with atrial fibrillation; however, concern over falls and subsequent bleeding may lead to under‐prescribing. Implementation of the HAS‐BLED and CHA_2_DS_2_‐VASc scores may help guide pharmacotherapy decision‐making.^44^, ^45^ Newer agents such as the direct oral anticoagulants (DOACs) are now considered the preferred option demonstrating similar efficacy in preventing stroke and systemic embolism, with a lower risk of major bleeding compared to warfarin.^46^ Warfarin also seems to be associated with increased risk of osteoporotic fractures compared to the DOACs.^47^ All DOACs require dose adjustments for renal function. Apixaban is often considered the preferred agent for older adults, as it has lower renal excretion than dabigatran and rivaroxaban. For those with end stage renal disease, either apixaban or warfarin is reasonable.

*Rate vs rhythm control*: Symptomatic management of AF consists of either rhythm control or rate control. Rhythm control was shown to be inferior to rate control with respect to mortality in older adults and is associated with more adverse drug events and hospitalizations.^48^ Moreover, antiarrhythmic agents use is limited in this population due to co‐morbidities including structural heart disease, heart failure, and renal dysfunction. Amiodarone may often be the only appropriate option, although its use is associated with many long term side effects. Once AF becomes permanent and the decision to pursue rate control is made, all antiarrhythmic agents should be discontinued. Rate control is usually achieved with beta‐blockers, or a nondihydropyridine calcium channel blocker (CCB) such as diltiazem.^49^ Atenolol should be avoided in this population due to its renal excretion. Digoxin may also be used for rate control when hypotension limits beta‐blocker or CCB use, but it requires strict monitoring to ensure therapeutic drug levels. Chronic digoxin use appears to be associated with increased mortality.^50^ Therefore, initiation of digoxin therapy in older adults with atrial fibrillation alone should generally be avoided when possible.

## 2 CONCLUSION

The impact of polypharmacy in older adults can range from reduced quality of life (pill burden, drug cost) to serious adverse drug events (side effects, toxicity due to decreased metabolism, drug‐drug interactions). Most would agree that striking a balance between over‐ and under‐treatment is important, yet doing it effectively is challenging due to the lack of clear guidance and insufficient clinical experience. When assessing whether or not to deprescribe, decisions should be individualized with an emphasis on shared decision‐making with the patient and family, if at all possible. Each time a provider comes in contact with an older adult is an opportunity to review current medications and assess the risk and benefit of each therapy.

## CONFLICT OF INTEREST

The authors declare no potential conflict of interests.

The authors would like to thank Meredith Azeem Pharm.D. for constructive criticism of the manuscript.
